# The ethics of clinical trials

**DOI:** 10.3332/ecancer.2014.387

**Published:** 2014-01-16

**Authors:** Cecilia Nardini

**Affiliations:** Foundation and Ethics of the Life Sciences (FOLSATEC), Department of Health Sciences, University of Milan, Italy; European Institute of Oncology (IEO), via Adamello 16 20139, Milan, Italy

**Keywords:** ethics, RCTs, research, personalized medicine, precision medicine

## Abstract

Over the past decades, randomised controlled trials (RCTs) have prevailed over clinical judgement, case reports, and observational studies and became the gold evidential standard in medicine. Furthermore, during the same time frame, RCTs became a crucial part of the regulatory process whereby a new therapeutic can gain access to the drug market. Today, clinical trials are large and tightly regulated enterprises that have to comply with ethical requirements while maintaining high epistemic standards, a balance that becomes increasingly difficult as the research questions become more sophisticated. In this review, the author will discuss some of the most important ethical issues surrounding RCTs, with an eye to the most recent debates and the context of oncological research in particular.

## Introduction: research and experimentation on human subjects

Research involving human subjects has anything but a glorious legacy. The term ‘human experimentation’ still evokes, in many, the ghastly impression of the infamous experiments conducted on war prisoners during World War II. Furthermore, this negative impression was propagated in the postwar period by some notable cases of unethical handling of human subjects in medical research—episodes involving prisoners, the mentally disabled, the poor, or ethnic minorities, such as, for instance, the ill-famed Tuskegee syphilis study [1].

Such episodes, taking place in democratic and civilised countries, were the proof that war atrocities were not the only threat to the condition of human research subjects: the conception of research ethics had to be recast as a whole. Indeed, until as recently as the 1970s, the medical investigator was considered the sole authority that could adjudicate the legitimacy of a study protocol. The protection of participating patients was generally considered to be warranted by the commitment of physicians, by the Hippocratic Oath, to ‘do no harm’ to their patients. The necessity of a research ethics distinct and independent from medical ethics emerged only in the moment these episodes of research misconduct exposed such conviction in all its inadequacy. The endeavour of medical research actually confronts physicians with an ethical dilemma. On the one hand, the doctor is bound by her professional ethics to do all that is in her power to benefit her current patient. On the other hand, though, the doctor has also an obligation to forward medical science to the benefit of future patients. The necessity of a framework for critically discussing and evaluating human experimentation arises because the tools of medical ethics alone are insufficient to direct a course of action in the face of such a dilemma. A physician who is personally more inclined towards scientific progress may feel that her duty falls more on the side of pursuing research and thus eventually establishing better therapeutic options, while her colleague may instead feel bound to care for her current patients regardless of medical progress. Furthermore, in such a framework, there is no place for considerations that we do instead value in other contexts in our society, such as the right of patients to decide whether they want to take part in research or not.

Our modern concept of research with human subjects is inspired by three influential documents, conceived in the aftermath of the episodes of research misdemeanour, which were mentioned in the beginning. The Nuremberg Code [2] is a legal and ethical code promulgated by the U.S. judges at the trial of the Nazi doctors at Nuremberg after World War II. Many consider it as the most authoritative legal reference on the subject of human experimentation. It is based on universal principles of natural law and human rights, and it establishes the basic principle that the participation in research requires the free, informed consent of the participating subject. The Declaration of Helsinki [3] is arguably the most widely known and influential guideline in medical research worldwide. It is an official policy of the World Medical Association (WMA), which was adopted for the first time in 1964 and has since undergone a number of revisions. The Declaration can be regarded as the expression of the WMA’s effort in balancing the need to generate sound medical knowledge with the need to protect the health and interests of research participants. Finally, the Belmont Report [4] is a short document on moral principles that was published in 1978 by the National Commission for the Protection of Human Subjects of Biomedical and Behavioral Research, in the aftermath of scandals of research misconduct that were uncovered in the 1970s. The Belmont Report is especially known for establishing a framework of basic moral principles—respect for persons, beneficence, and justice—which should guide the conduct of research. At the European level, further guidelines are provided by the directives of the Council of Europe and the European Commission, and of course, by the individual member States’ National Bioethics Commissions. This review focuses on a subset of research involving human beings, namely the stage of clinical research in which a new therapeutics or medical intervention is put to test on human patients: the clinical trial.

## The randomised controlled trial

The randomised controlled trial (RCT) consists, in its most conventional form, in a comparison of the action of the experimental treatment versus the untreated progression of an illness under study. The comparison takes place under tightly controlled conditions in order to extrapolate a generalisable conclusion from the study.

From the epistemic point of view, the idea behind the RCT is that of the counterfactual analysis [5]. When a new treatment is administered to a patient and an improvement in her condition is observed, the possibility of drawing a conclusion from the fact is hindered by the absence of a counterfactual: possibly the patient would have recovered anyways if left untreated, or maybe a different treatment would have been more effective. In an RCT, participants are divided into two groups, one that receives the experimental treatment and another that acts like a control, providing the answer to the ‘what if’ counterfactual question. For the concept to work as intended, though, the administration of the experimental treatment should be the sole difference between the experimental and the control group. In actual trials, this is clearly an idealisation: internal variability of groups and contingent differences between groups will introduce confounding factors that affect the possibility to draw conclusions from a trial.

Several aspects of the scientific design of trials have precisely the objective of minimising this kind of interferences on the results. One of the most notorious such aspects is randomised allocation of subjects, typically associated with blindfolding of participants and possibly also of investigators. Patients entering a trial are assigned to either the experimental or the control group following a non-predictable, chance-based procedure, and neither they nor the investigators and the participating physicians know to which arm they have been assigned. This procedure has the primary objective of removing subjective interferences, for instance, the possibility that investigators assign healthier patients to one arm of the study to begin with [6]. Analogously, the methodology of the statistical test of significance is used as an impartial way to distinguish genuine differences in treatment effectiveness from occasional fluctuations in patients’ response to treatment, even though the adequacy of the significance test to this task is a subject of much methodological controversy [7]. Because of its scientific credentials, the RCT methodology is currently considered the gold standard in treatment evaluation. Over the past several decades, RCTs prevailed over clinical judgement, case report, and observational studies as evidential standards in medicine, largely due the effort of the movement known as evidence-based medicine [8]. Furthermore, during the same time frame, RCTs became a crucial part of the regulatory process whereby a new therapeutic can gain access to the drug market. Nowadays, clinical trials are large and tightly regulated enterprises that have to comply with ethical requirements while at the same time maintaining high epistemic standards, in a balance that becomes increasingly difficult as the research questions become more sophisticated.

## The main ethical issues surrounding RCTs

The general problem with the ethics of clinical trials stems from the fact that those who stand to gain from the trial results are not the same that bear the risk and burden of trial participation. Participation in a clinical trial entails an increased level of risk with respect to ordinary clinical care, particularly due to the potential for exposure to unexpected effects of a new treatment. These risks are actually not offset by a prospective clinical benefit, since the primary end of the trial is not that of treating trial participants but rather that of producing generalisable medical knowledge. In the following, we will see that this ethical tension has several facets, according to which aspect of the RCT is put in the spotlight. For each of the problematic aspects that will be examined—informed consent, use of placebo, randomisation, and protection of participants—the author will provide a brief introduction, then present the ethical principles and concepts that come into play, and finally discuss the most relevant issues that are still open on the subject.

### Participation and informed consent

The past history of medical research features several episodes in which the burdens of research participation were placed disproportionally on trial participants, either by deceiving them with the promise of a cure or by deliberately concealing that they were taking part in research. In our modern ethical conception, this is no longer considered acceptable, and all research conducted on human subjects must be pre-emptively accepted by the subject themselves through the procedure known as informed consent. One of the most important ethical constructs of modern biomedical ethics, informed consent is nowadays an essential condition both for therapy and research. Written authorisation forms were occasionally submitted to participants also in the early times of medical experimentation. However, this was, in most of the cases, a device aimed at ensuring the subject’s compliance rather than an expression of concern for their welfare. Modern informed consent is very different from these early instances in that it stems from a basic principle, expressed in the Nuremberg Code, of the respect due to persons and the value of a person’s autonomy.

In the modern conception, consent to a therapy or a research protocol must possess three features in order to be valid. It should be voluntarily expressed, it should be the expression of a competent subject, and the subject should be adequately informed. Even though all the three components of informed consent have problematic aspects in their empirical application, the notion of informed consent is most often put to question because of the difficulty in specifying what is an adequate level of information for the consent to be valid [9].

Information that makes consent valid is generally thought to include the understanding of the risks and benefits of the treatment(s) that patients may receive, understanding of the procedures that the participant may undergo, including, in the case of RCTs, also blinding and randomisation, understanding that participation in research is voluntary, and finally understanding of the *purpose *of the research. What counts as an adequate level of information at each of these stages is notably difficult to define. In part, this issue presents itself also in the context of medical care, where informed consent of the treated subject is necessary for performing any diagnostic or therapeutic intervention. This means that the patient should have an understanding of the diagnostic or therapeutic procedure, its risks and prospective benefits; something which is clearly challenging for the MD to provide in case of complex procedures [10].

In medical research, however, the issue has an additional twist due to the fact that the aim of research is not the direct benefit of research participants. Despite the chance that participating patients might receive a therapeutic benefit while enrolled in a trial, this is not the primary end of the trial. Participants may fail to recognise that the purpose of the study is not to find the best therapy for *them*, thus falling in what is called the *therapeutic misconception *[11]. Some medical scholars maintain that the therapeutic misconception is actually encouraged by ‘a predominant ethical view’ that medical investigators, since they are also medical doctors, should conduct their research with therapeutic intent [12]. According to the said authors, this attitude is misguided and it is potentially conducive to the exploitation of participants.

### Use of placebo and deception

Personal expectations about treatment entertained by both patients and investigators may play an unexpectedly large role on the progress of a therapy. For this reason, the scientific test of a new intervention may require that patients in the control group receive a placebo. Placebos are interventions that lack the active principle of the experimental treatment but that are otherwise indistinguishable from it.

A first issue concerning the use of placebos concerns the problem of deception. Patients on the placebo arm of a clinical trial must be made to believe they are receiving a working treatment, even though they are not, for the placebo effect to play a role at all. Despite the appearances, however, it is contentious that placebo-controlled trials (PCTs) are inherently deceptive towards participants. This is because participants are actually informed, as they consent to the study, that they will not be told whether they are receiving active medication or placebo [13].

There is, however, a most serious issue with the use of placebo, i.e., the possibility that participants are *harmed *by receiving a placebo instead of an active treatment. For many conditions, not receiving an active treatment exposes the patients to higher levels of pain, an aggravation of their conditions, or even the risk of death. In such situations, clearly, the use of placebos is downright unethical, because the patients on the placebo would be harmed for the sole benefit of third parties—namely for the scientific achievement of the trial completion. In some such cases, the traditional placebo-controlled design can be modified so to use the same population of patients to study both the placebo response and the response to the active treatment, thus avoiding that some in-trial patients are left untreated. This kind of trial is called a *crossover *trial because patients in the trial cross over at predetermined time points from the placebo to the treatment arm and vice versa [14]. Another option is the ‘add on’ design, in which both groups receive the standard treatment plus either the experimental treatment or the placebo.

However, these non-conventional design options are not applicable in all situations; more importantly, in some cases, measuring the effectiveness of the new treatment as compared with placebo is less relevant than confronting the new treatment, within a clinical setting, with the established standard treatment for the illness under study. In such cases, there is the possibility to conduct an active-controlled trial (ACT). Even though it might appear that there is a clear ethical and scientific rationale to the use of active controls in place of placebo controls, the conduct of ACTs is nonetheless controversial. In fact, it has been suggested [15] that ACTs, unlike PCTs, cannot effectively discern differences in effectiveness between treatments due to the lack of the ‘zero point’ reference mark provided by the placebo control. This issue, known as the problem of *assay sensitivity*, is a subject of critical discussion [16, 17].

In most of the ethical regulations and research guidelines, the use of placebo controls is subjected to a delicate tradeoff between the stringency of the scientific rationale for using it, and the possibility of harm for participating patients. For instance, according to the Declaration of Helsinki in its most recent formulation, the use of placebo is acceptable under the condition that no proven treatment exists, but also ‘where for compelling and scientifically sound methodological reasons, [it] is necessary to determine the efficacy or safety of an intervention’, provided that ‘the patients who receive placebo or no treatment will not be subjected to any risk of serious or irreversible harm’ [3, art 32]

### Randomisation and blinding, and equipoise

RCTs involve, by definition, randomisation and often blindfolding of participants. These two epistemic devices are needed in order to rule out the most obvious perturbations of the trial result due to the interference from the investigators or the patients themselves. However, randomisation and blinding may come to conflict with the individual interests of those participating in the trial. A first reason for this conflict is that, when randomisation and blinding are in place, patients cannot enjoy individualised treatment decisions responding to their condition [18]. This, however, is something patients explicitly consent to when they endorse trial participation. Randomisation between the two arms of the study does, however, raise a further ethical concern that is not so easily dismissed. By entering an active-controlled RCT, participating patients stand a chance of receiving the treatment that will eventually turn out to be inferior. This is especially problematic if the experimental treatment proves to be worse than the standard that was available outside of trial, since it is a recognised ethical principle that patients should receive the best proven standard of care whenever feasible (see [3]). Apparently, then, randomisation harms trial participants that, by entering the trial, may be denied the best standard of care available.

The view that is currently prevalent in the ethical literature is that *equipoise*, denoting an epistemic state of indifference between two treatments, can relieve this ethical tension. If the medical community is in equipoise, this means that there exists a state of ‘honest professional disagreement’ among medical scientists about which treatment is best [19]. In this situation, randomisation does not harm trial participants because it constitutes a ‘fair bet’ procedure among outcomes that are *a priori* equally valuable [20].

Notwithstanding its success as an ethical paradigm, however, equipoise has critics. A first problem is that of identifying under which conditions equipoise is present given a particular clinical question [21]. Furthermore, an all but obvious point is *whose *equipoise or indifference should be morally relevant. While the currently predominant notion, based upon considering the state of knowledge of the scientific community, appears as the most reasonable choice, other options may have a sound rationale as well. For instance, at least for some conditions, equipoise of the participating patients should be just as relevant, since we can hardly expect a patient to be indifferent between, say, an invasive surgical procedure and a therapy based on oral drug administration [22]. Despite the problems just mentioned, however, equipoise remains a workable ethical paradigm for adjudicating the ethics of clinical trials, routinely used by ethical boards in research hospitals in taking decisions about the approval of new studies.

It is worth mentioning, in closing the section, that medical investigators and biostatisticians in the past have strived for finding a methodological solution in order to minimise the chance that patients are exposed to the less effective treatment. Techniques such as unequal randomisation—i.e., randomisation with rates different from 50–50—or adaptive randomisation—where allocation rates vary with the trial results as they accumulate, favouring the treatment which is proving more effective—have been proposed and occasionally used in the past to this aim [23]. However, such methodological solutions pose more ethical problems than they solve, given the difficulty of justifying the ethics of enrolling in the trial the patients that end up in the non-preferred arm.

### Navigating between exploitation and overprotection of patients

As discussed in opening the section, in clinical research, there exists a gap between those who are exposed to the risk of a medical intervention—the trial participants—and those who are the intended beneficiaries of the trial results—future patients and society at large. The existence of this gap has informed the conception of most ethical guidelines that are currently in use, which were created with a keen eye to protecting participants from the risks and the burdens of research. As an instance, the Helsinki Declaration requires that ‘the wellbeing of the individual research subject must take precedence over all other interests’ [3, art 6]. In recent years, however, this paradigm of emphasised participants’ protection is increasingly considered inadequate. Mostly, two considerations speak against it.

The first point is the observation that the sole effect of such strict regulation in developed countries has been that of encouraging the outsourcing of trials conduct to countries where standards for the protection of participants are lower. This is clearly an issue, also due to the fact that both the national states involved and the prospective participants individually often find themselves in a situation of economic vulnerability and captivity towards the large pharmaceutical groups that are running the trial (see [24] and [25]). Thus, strong protection norms prove ultimately ineffective in warranting high levels of protection to participants in a globalised setting, appearing on the contrary to foster new forms of exploitation. Negotiating the adequate level of protection that can be set as a global standard for medical research has proven challenging, as testified by the continuing effort in revising of the Helsinki Declaration [26].

A second argument that has been raised against the current paradigm concerns the issue of paternalism [27], i.e., the concern that the levels of protection that are warranted by current guidelines may conflict with the autonomous choices of participants. A patient participating in a trial might wish to take a higher level of risk for the sake of an individually gauged perceived benefit, for instance, by taking a chance with an innovative and promising treatment. Or, more controversially, a patient might wish to take part in a research from which she knowingly stands no chance of receiving any benefit, for the sake of benefiting other patients or posterity. This latter is the case of so-called ‘Phase 0’ trials in oncology, pilot studies conducted on terminal patients in absence of any therapeutic expectation [28]. Current regulations seem incompatible with this view because of the emphasis they put on protection of participants; this unless the notion of ‘well-being’ of the participating subjects is interpreted fairly liberally to include the notion of fulfilling one’s desire to help others or forward medical progress [29].

## An outlook on oncological research

The issues discussed in this review stem from general features of clinical research and the RCT methodology, such as the need to subject patients to blindfolding and random allocation between treatment arms, or the point that medical research and practice respond to different ethical standards. In closing this review, the author will examine the context of oncological research more specifically. In all its forms, cancer is a dangerous disease that puts a threat on the patient’s life. As a consequence, most of the ethical issues we discussed through this chapter—for instance, the legitimacy of the use of placebo or the risk of therapeutic misconception—arise in the context of testing anticancer drugs to a preeminent point. (Surgical procedures will not be considered in the following discussion, even though they represent the front-line intervention against several tumours, due to the fact that they are not typically evaluated through RCTs [30].)

In addition to the questions already explored through the review, however, oncological research presents a whole new family of ethical issues as the field goes through the so-called genomic revolution. The completion of the Human Genome Project has brought about the potential for a profound transformation of the understanding and managing of non-infective diseases. The offshoot is *personalised *or *precision *medicine, or the idea of proceeding from the genetic and molecular hallmarks of common diseases in order to design and administer situationally the least harmful and most effective treatment [31]. Molecular biology, and the quest for molecularly targeted agents, are playing an increasingly major role also in oncological research [32]. While traditional anticancer agents target, in a non-specific manner, all fast-dividing cells, novel molecularly-based agents are expected to act in a selective manner on precise nodes of cellular pathways that are mutated or dysregulated in cancer cells. The two most renowned of these compounds are Gleevec (imatinib) in chronic myelogenous leukaemia (CML) and Herceptin (trastuzumab) in breast cancers characterised by overexpression of HER2 receptor.

Targeted cancer therapies give doctors a better way to tailor cancer treatment, especially when a target is present in some but not all tumours of a particular type, as is the case for HER2. Eventually, treatments may be individualised based on the unique set of molecular targets produced by a patient’s tumour. Targeted treatments also hold the promise of being more selective of cancer cells versus normal cells with respect to traditional chemotherapeutics, thus harming fewer healthy cells, reducing side effects, and improving the quality of life. Targeted treatments represent the major way forward in oncological research, and they are a solid clinical reality for the management of some disease subclasses, such as the already cited CML.

Despite this, the development and use in the clinics of targeted agents is as yet underexplored in its ethical consequences. From the point of view of the clinical practice, the most serious issues are raised by concerns of distributive justice, especially face the escalating costs of molecular agents [33]. However, the focus of this review is the context of clinical research, and of clinical trials in particular. As a matter of fact, the testing of targeted agents is an even more neglected topic in the ethical literature; in the following, the author will present some observations based on original research. The reader should be warned, though, that the complexity and breadth of the issues involved far exceeds the scope of this review.

### Testing targeted agents: some ethical considerations

Targeted agents present a peculiarity that puts them apart from other anticancer drugs: the selective nature of the drug’s action. When patients that have the same kind of tumour, but harbouring different molecular lesions, are exposed to a targeted compound, the response can vary dramatically to the point that not only the magnitude, but also the direction of the treatment effect may be different across molecularly identified subgroups. This is particularly relevant for the testing of such drugs, as it means that the beneficial effect of the targeted agent in trial is often restricted to a small class of the initially eligible patients, and the class of patients that would benefit often cannot be determined prior to beginning the study [34]. Thus, in a study testing a targeted agent, only a small fraction of the participating patients have a prospect to benefit from the experimental treatment at all. While apparently this seems true of conventional RCTs as well, a closer inspection reveals the ethical issue as specific of targeted therapies. In the case of a treatment that has universal application, like a conventional cytotoxic agent, whether the trial is successful or not, it will have established a conclusion that is relevant also for the patients that were participating—this prospectively justifies their participation. However, this is admittedly not the case for participants in a targeted therapy trial—at the outset of the trial, it is known that the conclusion about the new therapy will at best have a relevance only for a small fraction of these patients. This unprecedented ethical issue can indeed be alleviated in case there exist means—such as reliable tests or biomarkers—to single out for trial participation only those patients which are likely to respond; at the time being, however, this is unfortunately not often the case.

Furthermore, the possibility of conducting trials only on a highly selected population of patients raises a new set of concerns. It has been argued that precision medicine calls for a shift in the drug testing paradigm, from the current one based on large RCTs and the centrality of statistical evidence, to one based on shorter, smaller trials that combine the statistical evidence from the trial with causal knowledge coming from the lab [35]. *Prima facie *such a shift would be beneficial in terms of the ethics of the targeted trials, since small trials expose only a minimum number of patients to the controversial risks and burdens of trial participation. Indeed, there seems to be a strong ethical rationale in having trials involve the least number of patients necessary to achieve a conclusion. However, the issue is with the reliability of the conclusions that can be arrived at through such trials. If the trial is designed in a way that prevents the achievement of its ultimate scientific purpose, i.e. reliable and generalisable medical knowledge, participation of human subjects to the trial endeavour fails to be justified. Underpowered trials—trials with insufficient participants to achieve adequate statistical power—have repeatedly been denounced as unethical on these grounds [36], and recent work shows [37] that the same argument could indeed be raised against small, biomarkerdriven trials of targeted therapies.

The ethical issues just presented in relation to the testing of targeted therapies seem ultimately to boil down to a problem of conflicting evidential standards. What needs to be resolved is whether the evidence to prove a personalised therapy efficacious differs, and in what ways, from the evidence that is needed to put conventional medicines to test. Only when this epistemic point is clarified, it will be possible to qualify the ethical issues discussed above and possibly solve them. Indeed, as philosopher of medicine John Worrall has argued, ‘no informed view of the ethical issues… can be adopted without first taking an informed view of the evidential-epistemological ones’ [38]; this view seems to apply particularly well to the case in point. Thus, it seems that the ethics of targeted trials cannot be properly adjudicated until the evidential peculiarities of personalised therapies find a firm place in the evidential paradigm of medicine. Whether this requires a paradigm shift or just a small adjustment is, however, an entirely different and still open question [39].

## Conclusion

The distinctive issues that are faced as oncology transitions to a precision medicine paradigm, as well as the other issues analyzed in this review, testify that the ethics of clinical trials becomes increasingly complex to evaluate as clinical research progresses, as research questions become more sophisticated, and as the research context as a whole grows to an increasing level of interplay among diverse actors. The ethical discourse has to keep abreast of these changes, in order to provide an adequate guidance for medical research in the future.
